# Avian Influenza A Viruses Modulate the Cellular Cytoskeleton during Infection of Mammalian Hosts

**DOI:** 10.3390/pathogens13030249

**Published:** 2024-03-14

**Authors:** Flora De Conto

**Affiliations:** Department of Medicine and Surgery, University of Parma, Viale Antonio Gramsci 14, 43126 Parma, Italy; flora.deconto@unipr.it

**Keywords:** avian influenza A virus, pandemic, zoonosis, pathogenicity, mammalian adaptation, host species barrier, cytoskeleton

## Abstract

Influenza is one of the most prevalent causes of death worldwide. Influenza A viruses (IAVs) naturally infect various avian and mammalian hosts, causing seasonal epidemics and periodic pandemics with high morbidity and mortality. The recent SARS-CoV-2 pandemic showed how an animal virus strain could unpredictably acquire the ability to infect humans with high infection transmissibility. Importantly, highly pathogenic avian influenza A viruses (AIVs) may cause human infections with exceptionally high mortality. Because these latter infections pose a pandemic potential, analyzing the ecology and evolution features of host expansion helps to identify new broad-range therapeutic strategies. Although IAVs are the prototypic example of molecular strategies that capitalize on their coding potential, the outcome of infection depends strictly on the complex interactions between viral and host cell factors. Most of the studies have focused on the influenza virus, while the contribution of host factors remains largely unknown. Therefore, a comprehensive understanding of mammals’ host response to AIV infection is crucial. This review sheds light on the involvement of the cellular cytoskeleton during the highly pathogenic AIV infection of mammalian hosts, allowing a better understanding of its modulatory role, which may be relevant to therapeutic interventions for fatal disease prevention and pandemic management.

## 1. Introduction

The *Orthomyxoviridae* family includes seven genera and nine viral species. The viral species belonging to the genera *Alphainfluenzavirus*, *Betainfluenzavirus*, and *Gammainfluenzavirus* can infect humans and other animals. Each of these genera includes one viral species (influenza A virus, influenza B virus, and influenza C virus, respectively), based on antigenic differences in the ribonucleoprotein and matrix protein antigens. Influenza A and B viruses exert a more relevant pathogenic role in humans. Moreover, both of them cause seasonal epidemics of influenza, while only influenza A viruses (IAVs) are known to have caused several pandemics in the past with enormous mortality and economic costs [1,2]. 

The World Health Organization (WHO) estimates that there are around a billion cases of seasonal influenza annually, including 3–5 million cases of severe illness. Seasonal influenza may result in 290,000–650,000 deaths yearly due to respiratory diseases alone [3]. For the prevention of influenza virus infection, vaccine administration to people aged 6 months and older is fundamental and can lessen the severity of infection. The vaccination is formulated every year because of antigenic drift (mutations), which allows the influenza viruses to escape the pre-existing immunity. However, the vaccination is effective only when the vaccine is well-matched to circulating influenza virus strains. Moreover, it has been assessed that influenza viruses can develop drug resistance and escape selection pressure [4].

IAVs are enveloped, negative-sense, eight single-stranded, segmented RNA viruses that replicate in the nucleus of infected cells using their viral RNA polymerase. IAVs are respiratory pathogens causing a severe threat to human health worldwide, mostly due to their large genetic variability and ability to cross host species barriers [5]. IAVs naturally infect various avian and mammalian species with different biological features. Direct droplet spread is the most common method of influenza transmission and the incubation period is about two days. Influenza infections in temperate climates tend to occur most frequently during midwinter months. 

IAVs have been divided into different subtypes based on the antigenic features of the surface glycoproteins, hemagglutinin (HA or H, 18 subtypes) and neuraminidase (NA or N, 11 subtypes), which exhibit much greater amino acid sequence variability than in influenza B and C viruses. IAV subtypes endemic in animals include H1N1 (pig), H3N2 (pig, dog), H3N8 (horse), and H5N1 (bird) [6]. Wild aquatic birds are considered the main structural reservoir of H1 to H16 IAV subtypes [7,8,9]. Conversely, the subtypes H17N10 and H18N11 have been found only in bats. Specifically, 144 avian influenza A viruses (AIVs) in different subtypes can be found in infected birds, which may spread influenza viruses through mucus, saliva, and feces [9]. In general, human influenza viruses are sensitive to heat, acidic pH, and solvents, while avian strains survive longer in the environment.

Although in the literature there is a wide availability of data on the characteristics of IAVs, an accurate in-depth analysis of the mammalian host factors co-opted during AIV infection is lacking. This review aims to focus on the involvement of the cellular cytoskeleton during the infection of mammalian cells by AIV strains. The data presented may constitute a useful premise for future in-depth studies and the possible discovery of innovative therapeutic approaches. 

## 2. Avian Influenza A Virus Infection Transmission to Humans

AIVs are globally challenging due to widespread circulation. They are differentiated into low- and high-pathogenic viral strains. Low-pathogenic viral strains only cause mild illness in domestic birds. Conversely, highly pathogenic avian phenotypes, such as H5N1, H5N8, and H7N9, may cause organ damage, affect the respiratory tract, and determine up to 100% bird mortality [10,11]. Generally, highly pathogenic AIVs emerge from low-pathogenic viruses, acquiring cleavage site insertions that promote systemic infections [12]. 

AIVs show the potential to be episodically transmitted from birds to other animals and humans by direct contact or through an intermediate host. AIV infections in humans range in severity from no symptoms or mild illness (fever, conjunctivitis, and mild influenza-like upper respiratory symptoms) to severe diseases (pneumonia, acute respiratory distress syndrome, multi-organ dysfunction, and encephalopathy) and death; less common infection signs include diarrhea, nausea, vomiting, and seizures [6]. Most serious illnesses and high mortality rates have been associated with the Asian lineages H7N9 and H5N1 [3,13].

Although AIVs spread from one infected person to close contacts, prolonged and unprotected contact is rare and generally limited to a few people, there is a global concern that highly pathogenic AIVs (H5/H7/H9/H10) are adapting to other species by acquiring mutations in animals or humans that support their cross-species transmission [5,14,15,16]. Notably, the segmented RNA genome allows IAVs from different species to mix their genes by reassortment, generating new viruses. Important, adaptation processes of AIVs are needed for their efficient transmission and replication in mammal hosts. This aspect first includes the receptor–binding specificity switch of HA to the mammalian sialic acid 2,6 galactose-type receptor [17]. Moreover, the presence of specific mutations in the viral polymerase complex is considered an important host range determinant [18,19]. In addition, the NA of AIVs contains a specific sialic acid-binding site that is absent in human IAV, affecting the catalytic enzyme activity [20].

Since 1996, 11 subtypes of AIVs have been assessed to spread directly from infected birds to humans. Importantly, infected humans can transmit influenza viruses to other animals (pigs, poultry, cats, and dogs). This phenomenon is called reverse zoonosis and may contribute to the further diffusion of influenza viruses [21].

The highly pathogenic H5N1 AIV subtype emerged in China in 1996, establishing sustained transmission in domestic poultry [22]. From 1997, H5N1 human infections occurred in Asian countries with high mortality levels [23,24]; the results of serological assays demonstrated the human-to-human virus spread [25,26]. In a second distribution wave in 2003, the H5N1 virus was disseminated in Asia and Europe through migratory birds [27]. From 2003, several human H5N1 infection episodes were reported worldwide, and, up to 2023, the WHO documented 457 fatalities [28]. Since 2021, the H5N1 virus caused several infections in wild carnivores, mink farms, and marine mammals [29]. The wider range of H5N1 infections and the involvement of different animal species have favored the emergence of new and potentially more virulent variants. Importantly, continuous genetic variability has been observed for the H5N1 virus across various geographic regions as well as increased virulence as it evolves [22,30,31,32,33,34]. In 2014, a fatal human infection by the novel H5N6 AIV was also reported [35].

H9N2 AIV has been circulating in poultry since 1994 and is occasionally transmitted to humans [36,37,38]. Importantly, the expanded receptor specificity of H9N2 has raised concerns as a possible source of a novel human influenza virus [39,40].

From 2003 to 2017, sporadic human infections caused by H5N1, H7N7, and H7N9 AIVs have been observed in different European and Asian countries [41,42,43,44,45,46]. Since 2013, newly emerging AIVs have frequently crossed the species barrier to infect humans, causing several fatal infections [47,48,49,50,51]. 

Overall, it has to be considered that both continuous influenza virus genetic evolution and the actual environment of human activities may facilitate the occurrence of zoonoses with a high transmissibility level, as evidenced by the recent SARS-CoV-2 pandemic. Moreover, highly pathogenic AIVs have never circulated extensively among humans; therefore, the lack of pre-existing immunity poses the human population with a high risk of severe influenza occurrence.

## 3. The Cellular Cytoskeleton

The cytoskeleton of eukaryotic cells is a well-organized and highly dynamic filamentous network that radiates through the cell and primarily comprises three filament types that function co-ordinately: actin filaments (microfilaments), microtubules, and intermediate filaments [52]. Septins are a family of GTPases and are considered the fourth cytoskeleton component [53]. Cellular motor proteins, such as dynein, kinesin, myosin, and other accessory proteins, represent a structurally and functionally diverse family of molecules that actively concur with cytoskeletal functions, facilitating the capture and transport of a variety of cargo along cytoskeletal networks. 

A complex cytoskeletal network connects the plasma membrane to the nucleus and carries out diverse roles [54]. Specifically, the cytoskeleton contributes to cell morphology regulation, cell migration, apoptosis, cell differentiation, and cell division [52,55,56,57,58,59]. Importantly, all cytoskeletal components can swiftly adapt to both external and intracellular stimuli, undergoing rapid and continuous structural modifications [60].

Microfilaments are thin and fibrous structures responsible for cytoplasmic streaming and represent the major structural component of the cell. They concentrate under the plasma membrane, are involved in cell division, maintain the plasma membrane structure, and participate in intracellular trafficking and cell motility. Several actin-binding proteins connect actin filaments, while other actin-binding proteins allow the interaction of actin with specific cytoskeleton components [61]. Microfilaments are considered the most dynamic cytoskeletal networks, as they can undergo rapid and significant depolymerization when cell deformation and movement are required [60,62]. Specifically, cortical actin is primarily implied in events related to the presentation of cell membrane molecules, endocytosis, and viral entry/exit mechanisms [63], while nuclear actin regulates chromatin remodeling and gene transcription mechanisms [64]. 

Microtubules are highly dynamic polymers of tubulin subjected to rapid cycles of polymerization and subsequent depolymerization, depending on the requirements of the cell [65]. Microtubules radiate from the centrosome, the microtubule-organizing center of eukaryotic cells [66]. They are involved in cell motility, signal transduction, the subcellular distribution of organelles, and intracellular transport [67]. Several microtubule-binding proteins regulate microtubule dynamics [68]. Both actin and microtubules form polarized filaments with growing plus-ends pointed toward the plasma membrane, allowing the ATP-mediated directional transport of cargo such as vesicles and organelles. Specifically, actin is generally involved in the short-range transport near the cytoplasmic membrane, while microtubules are for the long-range intracellular transport.

The largest gene family encodes the intermediate filaments (IFs). IFs are found in the cytoplasm and are adjacent to the inner face of the nuclear envelope. Comprised of more than 70 components, they show a remarkable diversity, and provide mechanical strength to animal cells, maintaining the cell shape and tension, and protecting the genome. Major IFs are classified into five types, based on their structural composition and origin: keratin, desmin, glial fibrillary acidic protein, vimentin, and neurofilament protein [69]. Keratins are the main components of the cytoskeleton in epithelial and mesenchymal cells. Keratins are classified into two groups, based on differential immunogenicity properties. They provide structural support to cells and counteract the effects of physical stress on cell integrity. Vimentin is assembled into major cytoskeletal systems in cells of mesenchymal and ectodermal origin. Recent evidence has shown that vimentin and filamentous actin form interpenetrating networks in the cell cortex and vimentin participates in the regulation of actin dynamics [70]. The intracellular distribution of vimentin allows the structural maintenance of cell organelles. Moreover, vimentin is an integrator of cellular mechanical processes, promptly responding to cellular stress [71].

Lamins are nuclear IFs and the principal component of the nuclear lamina, a mesh of proteins inside the inner nuclear membrane [72]. Lamins maintain the structural integrity of the nucleus and anchor the chromatin and nuclear pore complexes to the nuclear periphery [73]. In association with actin and actin motor proteins, these proteins mainly constitute the nuclear cytoskeleton [74]. Of note, they are involved in key roles, such as the regulation of nuclear morphology, stability, gene expression, and transmitting and deciphering mechanical stimuli into physiological responses.

Overall, the cytoskeletal scaffold regulates numerous aspects of cell biology, undergoing rapid and continuous structural changes to satisfy the requirements of the cell in both physiological and pathological conditions. Viruses represent an excellent tool to study the regulatory mechanisms of the cellular cytoskeleton. 

Viruses are obligate intracellular parasites that depend strictly on the host cell machinery to perform their successful replication [52,75,76,77]. Specifically, the cytoskeleton provides a scaffold for viral entry, sub-cellular trafficking through the dense cytosol, replication, assembly, and egression [78]. Viruses can subvert and exploit physiological cytoskeletal functions to promote their replication, with varying effects, depending on the virus species involved. The complexity of the cellular cytoskeleton and the differences between the replication cycles of human viruses belonging to different families allow very different types of interactions, which remain poorly described. The contribution of the cytoskeleton in the replication cycle of viruses is critical to expand our knowledge of virus-coopted regulatory mechanisms, which could allow the discovery of potential therapeutic strategies.

To date, considerable research activities have attempted to understand the pathogenicity and mechanisms of transmission of AIVs, focusing on the influenza virus themselves. Therefore, a comprehensive understanding of the response of host factors during viral pathogenesis in mammals is needed. In this way, characterizing the complex molecular mechanisms linking the cell cytoskeleton to influenza virus infection provides insights into the main filament network alterations, which might be useful for successful virus replication and transmission.

## 4. The Involvement of the Cellular Cytoskeleton in Influenza Virus Infection

In influenza virus infection, several cytoskeletal proteins exert a key regulatory role during internalization, endosomal acidification, and virus intracellular transport mechanisms [79,80]. It has been shown that endosomes containing influenza viruses co-localize with microfilaments and undergo further movements, suggesting the intervention of myosin motor proteins [81,82]. Myosin is also involved when influenza virus entry occurs through micropinocytosis [83]. In addition, keratin participates in the early cytoplasmic transport of influenza virus [84]. 

In the second step, the influenza virus intracytoplasmic transport switches from microfilaments to microtubules and the associated dynein motor protein, allowing the release of viral ribonucleoproteins near the nucleus, the site of viral genome replication [65,85,86]. It has been shown that intact microtubules promote both influenza virus entry to and exit from the host cell [82,87,88].

Vimentin regulates the activity and intracellular transport of IAVs, also exerting critical roles in the host cell response [80,89]. Specifically, it has been assessed that vimentin might play an important role in the regulation of lipids during H9N2 replication, providing an important antiviral target against the influenza virus [90]. Moreover, it has been shown that the integrity of IFs affects the release of H7N1 AIV progeny in mammalian cells, leading to decreased viral replication efficiency [91].

In the late phases of infection, the microfilaments regulate the distribution of newly synthesized influenza virus proteins at the plasma membrane, with the parallel involvement of myosin, contributing to viral assembly and budding [92,93]. 

**Figure 1:**
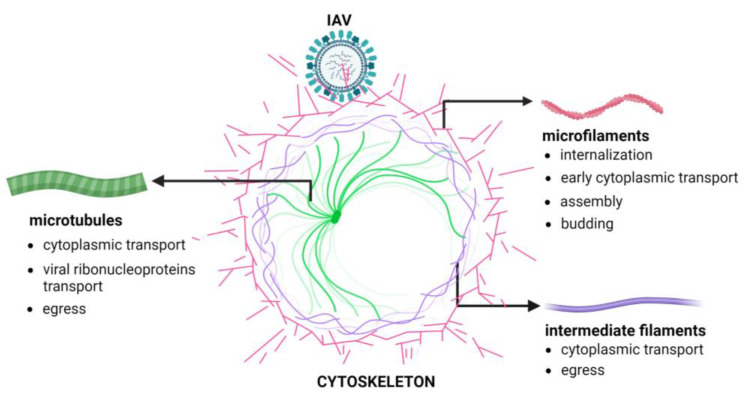
Cytoskeleton involvement during IAV replication. The Figure has been created with BioRender.com (accessed on 6 March 2024).

Figure 1 shows the phases of the IAV replication cycle requiring the active participation of the cellular cytoskeleton.

The cell-dependent highly dynamic or polymerized state of the cytoskeleton and the associated regulatory proteins may regulate the exit of influenza virus infection. In this regard, mammalian Diaphanous-related formin-1, a Rho-effector protein generating linear actin filaments and regulating microtubule organization, represents a restriction factor that counteracts cytoskeleton dynamics during the early phases of IAV infection [94]. In addition, it has been shown that highly polymerized/stable microfilaments and microtubules may restrict the early phases of IAV infection [81,91].

Notably, the influenza virus can modulate the dynamics of different cytoskeletal filaments by promoting their remodeling to support viral replication [95]. In this regard, the IAV can subvert the structural organization of cytokeratin 8, enhancing its phosphorylation state and thereby promoting its replicative efficiency [96]. Moreover, it has been assessed that the influenza virus stimulated the phosphorylation of cytoskeletal ezrin, radixin, and moesin, inducing structural cytoskeletal changes and permeability increases in pulmonary microvascular endothelial cells that favor its replication [97].

These observations strongly support the contribution of specific components of the cellular cytoskeleton to the replication of influenza viruses. Their intervention has some degree of variability, depending on both the cell type and the viral strain involved, attesting to the uniqueness of the influenza virus–host cell relationship.

## 5. Mammalian Cytoskeletal Proteins Are Modulated during Highly Pathogenic AIV Infection

The appearance of changes in the cytoskeletal protein pattern can affect the viral infection process [98]. Most of the studies on the cellular cytoskeleton’s role in viral infection have been performed using chemical compounds that depolymerize/disrupt a specific cytoskeletal component. Although the results obtained helped to identify the cytoskeletal pathways hijacked by viruses, the most relevant aspect that remains to be further dissected is the precise molecular mechanism underlying the interaction between the virus and specific cytoskeletal elements. 

Proteomic approaches reveal the differences between infected and healthy cells concerning specific proteins, as well as the occurrence of protein interactions and post-translational modifications. Another methodology that has been employed in this context is the weighted gene correlation network analysis, which partitions gene expression into groups of transcripts with highly correlated behavior, a seemingly strong predictor of related biological functions [99]. 

Studies relating to protein expression/structure modulation in mammalian hosts infected by highly pathogenic AIVs are very limited, likely because of the high-level biosafety measures required for experimental purposes. Therefore, this review aims to analyze the available results to stimulate the execution of future in-depth studies about the main cellular target proteins involved in mammalian adaptation to AIV infection. Moreover, the related signaling pathways of the activated host defense mechanisms should be dissected. 

Based on the available literature data, Table 1 shows the main cytoskeletal proteins that appear to be differentially modulated upon infection of diverse mammalian hosts by highly pathogenic AIVs. These findings suggest that specific cytoskeletal components are significantly involved in the pathogenic mechanisms of AIV infection at the cellular level, showing a fundamental understanding of underlying host targets.

Specifically, the most significant changes observed upon H9N2 AIV infection were identified in the upregulation of actin and keratin network expression in gastric adenocarcinoma and airway epithelial cells [37,100]. Interestingly, a profound reorganization occurring from the cleavage of actin and keratin networks suggests the possible virus-induced activation of reprogrammed host protein synthesis to favor viral protein expression [37]. 

In the case of H7N9 AIV infection, the downregulation of the F-actin capping actin protein was observed [101]. This event allows the microfilaments to keep growing, resulting in their abnormal accumulation inside the cell, therefore altering the regulation of different actin-binding activities, as well as affecting cell movement, cell polarity, and signaling events. 

With the H5N1 AIV strain, the expression of keratin, vimentin, and microtubules was upregulated for effective viral replication in human cells [99,102,105]. Conversely, the H9N2 AIV strain with E627K mutation in PB2, which has been associated with mammalian adaptation and increased virulence [18], downregulated the expression of several cytoskeletal and associated motor proteins [103]. 

Interestingly, the interaction between the nucleoprotein of different IAV strains (between two H5N1 AIV strains) and the cytoskeletal protein alpha-actinin-4 has been assessed by co-immunoprecipitation assays [106]. The authors of this study reported that during infection carried out with H1N1 IAV, the alpha-actinin-4 plays a crucial role in both regulating viral replication and viral nucleoprotein and/or ribonucleoprotein nuclear localization.

In summary, the analysis of the proteomic profiles of mammalian cells infected with different AIV strains reflects the specific host response to different pathotypes of influenza viruses. The results shown attest that specific cytoskeleton protein modulations were observed during highly pathogenic AIV infection. Importantly, the structure and functions of actin and keratin networks were mainly affected (see Table 1), given the key roles carried out by these cytoskeletal networks during viral infection. 

## 6. The Cellular Cytoskeleton Is a Mediator of Host Defense Responses during Influenza Virus Infection

Recent data provide evidence that the cellular cytoskeleton actively participates in the regulation of host immune response to viral infection, modulating the activation of the signaling pathway of interferons [107,108]. The cellular antiviral responses induced by type I interferons are considered the first line of host defense because the control of interferon production is critical for virus clearance. 

Viruses have evolved several mechanisms to exploit and remodel the host cell’s actin cytoskeleton at different stages of their life cycles. Consequently, the virus-induced actin disturbance is considered a priming signal for innate immunity activation [107]. Therefore, it appears conceivable that the cellular cytoskeleton carries out two strictly interconnected functions: it may both help viruses during their replication cycle and assist cells in activating the innate immunity mechanisms to provide rapid frontline defense [52]. The latter aspect is mainly achieved by using specific pattern-recognition receptors [109,110]. Specifically, viral RNAs are recognized by retinoic acid-inducible gene I receptors (RLRs), which stimulate the activation of signaling cascades leading to the release of cytokines and interferons [80,110]. 

The influenza virus modulates various host cellular processes to favor its replication and evade host responses. In this regard, specific cytoskeleton elements have been identified as mediators of host defense mechanisms [53,111,112,113,114]. 

Li et al. [99] showed atypical keratin gene expression during H5N1 AIV infection in association with the upregulation of inflammatory mediators. In this regard, the authors discussed the possibility that keratin molecules and their associated signaling patterns may contribute to shaping the defense mechanisms against viral infection.

Interestingly, evidence is mounting that vimentin plays an essential role in coordinating the signaling pathways that regulate inflammatory response mediator levels in viral infection and is a ligand for specific pattern recognition receptors (PRRs) [115,116]. Moreover, vimentin plays a fundamental role in coordinating the signaling pathways that regulate inflammatory response mediator levels in resident tissues, recruiting inflammatory cells from the blood [114]. Accordingly, it has been shown that transgenic mice defective for vimentin were less susceptible to mortality and tissue damage when infected with lethal doses of IAV, because of the decreased damage from inflammation [117]. In addition, it has been reported that vimentin can bind to nuclear factor kappa-light-chain-enhancer of activated B cells (NF-kB) sites, modulating intracellular signal transduction and immune response pathways [110]. Remarkably, vimentin can also be found at the cell surface or even secreted in macrophages and microvascular endothelial cells [118]. In this way, vimentin is exposed to external ligands, such as viruses, where it not only modulates the immune signaling pathways but also facilitates the cell entry of pathogens [119]. These data confirm that vimentin can be considered a negative regulator of innate antiviral immunity in case of both DNA and RNA virus infection [108]. Therefore, it appears conceivable that several pathogens can subvert vimentin functions. Accordingly, it has been assessed that vimentin expression rapidly increased in case of viral infection [102,113,118]. Moreover, the atypical activation of keratin gene expression was also observed upon H5N1 AIV infection, raising the possibility that keratin and associated signaling patterns contribute to shaping the host response [99,102].

Importantly, it has been shown that IAV can target and co-opt specific cytoskeletal components to subvert their role in inducing the antiviral response. In this view, the IAV PB1-F2 protein can translocate to mitochondria, accelerating mitochondrial fragmentation and impairing innate immunity [120]. More specifically, the microtubule-associated proteins 1A/1B light chain 3B have been identified as cytoskeletal interacting proteins of IAV PB1-F2 during its translocation to mitochondria. 

Although the influenza viruses first hijack the cellular cytoskeleton during different phases of their life cycle, modulating its structure and related functions for their productive replication, there is increasing evidence that this complex cellular component may promptly assist the host cell in the antiviral response [121].

## 7. Conclusions

Animal, human, and environmental health are strictly interconnected. Therefore, it is important to consider that more than 60% of human infectious diseases are derived from pathogens that originally circulated in animal species [122]. IAVs show very high genetic variability, facilitating their transmission and further adaptation to new hosts, including humans.

Both host and viral factors determine the virulence of highly pathogenic AIVs and their successful replication. However, the contribution of host factors remains largely unknown. Targeting cellular components instead of viral elements is more efficient for the search for novel therapeutic interventions that can broadly target viruses, considering the high level of variability of influenza viruses. 

The cellular cytoskeleton is a key player in a variety of cell features and functions and viruses can subvert its complex organization to create an environment that favors viral replication. This review focuses on the involvement of specific cytoskeletal proteins and their different modulations during highly pathogenic AIV infection of mammalian hosts. Although one of the limits of this review relates to the analysis of a small number of literature sources available about AIV infection and its relationship with the mammalian cytoskeleton, the findings shown could provide new insights and relevant information about the cellular pathogenesis of AIV infections. 

Importantly, increasing observations indicate that the cellular cytoskeleton may counteract viral invasion by activating the innate immune response through signaling regulatory molecules that induce rapid structural/functional rearrangements in its networks. 

A comprehensive understanding of cytoskeletal modifications and the host response during AIV pathogenesis provides new insights into the basis of interspecies transmission and mammalian adaptation, indicating the value of further investigation.

## Figures and Tables

**Table 1:** Differential modulation of mammalian cytoskeletal proteins upon highly pathogenic AIV infection.

Cytoskeletal Proteins	Main Roles	AIV Strain	Mammalian Host	Effects	References
actin	signaling, transcription, endocytosis, viral budding	H9N2	A549 cells human	upregulation	[100]
			AGS cells human	cleaved into fragments to reprogram host protein synthesis	[37]
F-actin capping protein	growth/polymerization of actin filaments	H7N9	A549 cells human	downregulation	[101]
keratin	structural support, communication between the plasma and nuclear membranes, interaction with small nuclear ribonucleoprotein bodies, metabolism, cell proliferation, apoptotic pathways, shaping host response	H5N1	Calu-3 cells human	upregulation	[99]
keratin 1,2,10			A549 cells human	downregulation	[102]
keratin 9,73,78				upregulation	[102]
keratin type I and II		H9N2	AGS cells human	cleaved into fragments	[37]
keratin 10			A549 cells human	upregulation	[100]
vimentin isoform 1	cell proliferation, differentiation, migration, signal transduction, and tissue remodeling	H5N1	A549 cells human	upregulation	[102]
dynein 1, ezrin, LIM, SH3 1, moesin	cytoskeleton regulation, cell shape control, modulation of signaling pathways, modulation of endothelial permeability	H9N2	mice	downregulation	[103]
myosin light polypeptide 3,4,9 and myosin light chain-2	motor proteins				
gamma-actin, keratin 78, septin-2 b, coronin actin-binding protein 1A, lamin B, tropomyosin alpha-1	gene transcription regulation, cell scaffolding	H5N1	mice	upregulation	[104]

Legend: human lung adenocarcinoma epithelial cells (A549); human gastric adenocarcinoma cells (AGS); human lung adenocarcinoma cells (Calu-3).
